# Mobility for sex work and recent experiences of gender-based violence among female sex workers in Iringa, Tanzania: A longitudinal analysis

**DOI:** 10.1371/journal.pone.0252728

**Published:** 2021-06-03

**Authors:** Zoé Mistrale Hendrickson, Anna M. Leddy, Noya Galai, S. Wilson Beckham, Wendy Davis, Jessie K. Mbwambo, Samuel Likindikoki, Deanna L. Kerrigan

**Affiliations:** 1 Department of Health, Behavior and Society, Johns Hopkins Bloomberg School of Public Health, Baltimore, Maryland, United States of America; 2 Johns Hopkins Center for Communication Programs, Johns Hopkins Bloomberg School of Public Health, Baltimore, Maryland, United States of America; 3 Department of Medicine, University of California, San Francisco, San Francisco, CA, United States of America; 4 Department of Epidemiology, Johns Hopkins Bloomberg School of Public Health, Baltimore, Maryland, United States of America; 5 Department of Statistics, University of Haifa, Mt Carmel, Haifa, Israel; 6 Department of Prevention and Community Health, Milken Institute School of Public Health, George Washington University, Washington, DC, United States of America; 7 Muhimbili University of Health and Allied Sciences, Dar es Salaam, Tanzania; Purdue University, UNITED STATES

## Abstract

Female sex workers are highly mobile, which may influence their risk of experiencing physical and sexual violence. However, there remains a paucity of research, particularly longitudinal, from Sub-Saharan Africa exploring mobility and gender-based violence among female sex workers. To address this gap, this study examined the longitudinal relationship between work-related mobility and recent experience of physical or sexual gender-based violence from a client or partner among female sex workers in Iringa, Tanzania. A secondary data analysis was conducted using baseline and 18-month follow-up data from Project Shikamana, a community empowerment-based combination HIV prevention intervention. Responses from 387 female sex workers aged 18 years and older participating in both baseline and follow-up were analyzed. Unadjusted and adjusted Poisson regression models with robust variance estimations, accounting for clustering of female sex workers’ responses over time, were fit. Final models adjusted for socio-demographic characteristics and aspects of participants’ living situations and work environments. Recent physical or sexual violence from a client or partner was common (baseline: 40%; follow-up: 29%). Twenty-six percent of female sex workers at baseline, and 11% at follow-up, had recently traveled outside of Iringa for sex work. In the final adjusted longitudinal model, female sex workers recently mobile for sex work had a 25% increased risk of any recent experience of physical or sexual gender-based violence when compared with their non-mobile counterparts (adjusted incidence rate ratio: 1.25; 95% CI: 1.03–1.53; p<0.05). Interventions must identify ways–such as mobile support services, linkages and referrals to health and other social services while traveling, or the use of mobile or digital technology–to address mobile female sex workers’ unique needs while traveling. Future quantitative and qualitative research is needed to understand the context of female sex workers’ mobility and how and why mobility influences risk environments and experiences of gender-based violence.

## Introduction

Female sex workers (FSWs) experience increased risk of physical and sexual violence globally [1,2]. Such gender-based violence (GBV) can be perpetrated by clients, non-paying or intimate partners, as well as fellow workers, police, and other individuals [2]. Experiencing violence can have additional negative consequences for FSWs’ mental and physical health, with demonstrated associations found across settings with reproductive coercion, inconsistent condom use, depression, and sexually transmitted infections including HIV infection [2–11].

The socio-structural environment in which FSWs live and work influences their risk of experiencing violence. Criminalization of sex work, gender inequities, inequitable power relations, economic constraints, and population mobility intersect to shape the contexts in which FSWs work and experience violence [2,11–15]. Population mobility, including both short-term and long-term mobility and migration, is an under-explored socio-structural factor relevant to FSWs’ risk of violence [2]. FSWs are often a highly mobile population, traveling for shorter and longer periods of time–both temporarily and more permanently–for diverse reasons and under different situations [16–18]. Such fluidity has led some researchers to consider different typologies to characterize mobile FSWs [18,19]. In Zimbabwe for instance, where mobility for sex work was reported by 59% of FSWs in 2016, Davey et al. (2019) identified distinct typologies of mobility based on destination, duration, frequency, and purpose of travel. These typologies suggest that mobility may have varying and nuanced effects on FSWs’ lives, work, and health. For example, work-related mobility may disrupt FSWs’ social relationships and limit social cohesion at their destination [20]. At the same time, travel for sex work can lead FSWs to work in venues or environments where they may have greater economic opportunities and earnings [16,21].

While substantial research has highlighted the increased risk for GBV among refugee and displaced women and girls [22–24], less attention has been given to the intersection of population mobility and GBV in FSWs’ lives more specifically [2]. A handful of studies have shown a positive association between recent mobility among FSWs and GBV [16,21,25–28]. Such mobility is often characterized as work-related and defined based on changes in workplace, working in multiple locations over a distinct period of time, engaging in contract work in a place away from one’s home, or traveling for the purposes of work to another district or region [16,21,25–28]. However, few studies have explored these relationships longitudinally or in diverse settings, instead relying primarily on cross-sectional data from South Asia and North America. One longitudinal study conducted with FSWs in Vancouver did find that those who had been mobile for the purposes of sex work anywhere outside of Vancouver faced an increased risk of violence over time from both clients and intimate partners [16]. Another study published in the same setting showed distinct changes in work-related mobility over time, with mobility marginally associated with harassment by police as well as concerns about safety [29]. However, these few longitudinal studies are limited to one high-income country and may not be generalizable to other contexts. As a result, there is an urgent need for additional longitudinal analyses conducted in other settings to inform public health programs and policies that acknowledge the intersections of mobility and violence in the lives of FSWs.

To fill these gaps, this article examines the longitudinal relationship between work-related mobility among FSWs and GBV in Iringa, Tanzania, where sex work remains criminalized [30]. In 2015–2016, 33% of FSWs had traveled outside of their district or the Iringa region in the past six months for the purposes of sex work [21]. A previous cross-sectional analysis showed that sex work-related mobility was positively associated with any recent experience of GBV as well as more severe forms of GBV [21]. This article builds on these initial cross-sectional findings to examine the longitudinal relationship between work-related mobility and GBV perpetrated by a client or intimate partner.

## Methods

### Ethics statement

This study was approved by the Johns Hopkins Bloomberg School of Public Health (JHSPH) institutional review board (IRB) (Ref #: 00007065) as well as the Muhimbili University of Health and Applied Sciences (MUHAS) IRB and National Institute of Medical Research (NIMR) IRB in Tanzania (Ref #: 1593). Oral informed consent was received from all participants. Oral informed consent was used given that the study worked with individuals with historically marginalized identities at the intersection of HIV- and sex work-related stigma. The study opted for oral consent because documenting their signature created additional possible risks related to participants’ confidentiality. This decision was informed by prior experience conducting the informed consent process for HIV prevention studies among people who sell sex and in the cultural context of Iringa. The informed consent procedures for this study were designed to maximize understanding of potential risks to participants. Forms were translated into local languages, and content read aloud to participants. Understanding was ensured by asking participants to summarize the study and explain the reasons why they wanted to participate. Individuals were also provided with a copy of their consent form if they wanted it as well as information on how to contact the study staff to report adverse events associated with their participation in the research. This oral consent procedure was approved by the JHSPH IRB as well as MUHAS and NIMR in Tanzania as part of the approval of the study.

### Study setting

This study draws on data collected from FSWs in Iringa, Tanzania, situated in the Southern Highlands of Tanzania. The Tanzania-Zambian (Tan-Zam) highway passes through Iringa, making mobility–be it by truckers traveling or residents–fundamental to life in this region [31–33]. Agricultural production is also an important aspect of the economy in Iringa, with residents and FSWs alike traveling seasonally for the harvest of crops like tomatoes, tea, and timber. Within this context, FSWs often travel along the Tan-Zam highway as well as seasonally for sex work [33]. The agricultural and transport industries both increase demand for sex work across various locations in and outside of the region, with a seasonal ebb and flow. A key type of clientele for FSWs along the highway is truckers; FSWs sometimes accompany truckers on spontaneous long-haul trips, with uncertainly about when and how they will return. As in other areas of Tanzania [34], FSWs in Iringa also travel to other areas–such as mining communities–for the purposes of sex work.

According to the 2016–2017 Tanzania HIV Impact Survey, HIV prevalence is higher in Iringa (11.3%) among adults 15 years and older than nationally (4.7%) [35]. Government HIV prevention programming in Iringa include HIV testing and counseling, both standalone and integrated into existing maternal and child healthcare services, as well as care and treatment services for people living with HIV [36]. Non-governmental organizations have worked in Iringa as well, with some services, including peer education and mobile outreach, tailored for women at increased risk for HIV, including FSWs [37–39].

### Study design and sampling

To examine the longitudinal relationship between sex work-related mobility and experience of GBV, this study drew on baseline and 18-month follow-up data collected as part of Project Shikamana. As described elsewhere [32,40], Project Shikamana was a prospective community-randomized trial conducted to examine the impact of a community empowerment-based combination HIV prevention intervention in two communities in the Iringa region of Tanzania. HIV prevention activities as part of Project Shikamana included mobile HIV testing and counseling, peer education, a drop-in center, and wider engagement with FSWs that focused on improving HIV service utilization, risk reduction, and fostering skills development and social cohesion [40,41]. One of the key components of this intervention focused on violence prevention, which was included at the request of women themselves based on their lived experiences [41,42]. Specific activities included workshops and trainings for FSWs as well as police focusing on how to prevent and respond to GBV. A working group was also formed for FSWs to share their experiences of violence. Resources, such as a safety tip card, were also developed to provide FSWs with information for how to prevent and report violence.

After a mapping of sex work venues in each community, time-location sampling was used to recruit both HIV-positive and HIV-negative FSWs for the baseline survey. Eligible FSWs were at least 18 years old and reported that they had exchanged sex for money, based on self-report, within the past month. Oral informed consent was received from all participants before beginning data collection.

Four-hundred ninety-six (496) FSWs were recruited at baseline, with 387 also participating in the 18-month follow-up survey. The final sample analyzed here included all FSWs participating in both baseline and follow-up surveys (n = 387).

### Measures

The outcome of interest was any recent experience of physical or sexual GBV from any sexual partner, defined as any self-reported experience of either physical or sexual violence within the past six months from a new or regular client or non-paying partner. Any recent experience of physical or sexual GBV from a sexual partner at baseline was assessed based on responses to nine questions following the WHO and work by Decker and colleagues [9,32,43]. At follow-up, questions were simplified and recent GBV was measured based on recent experience of physical violence–defined as being hit, slapped, kicked, pushed, shoved, or otherwise physically hurt–and sexual violence–forced sex by an individual or by a group–from new or regular clients or partners. This modification allowed the study to capture more accurately the forms of violence perpetrated by specific actors, rather than among all sexual partners (clients or partners). For the purposes of this analysis, recent experience of physical violence was defined, at both baseline and follow-up, if participants reported at least one form of physical or sexual violence from any sexual partner (clients or partners). The explanatory variable of interest was sex work-related mobility, defined as recent mobility outside of the Iringa region within the past six months specifically for sex work. Sex work-related mobility was measured at both baseline and follow-up. Refusal to respond was coded as missing for the purposes of analysis (n = 3 at follow-up only).

Confounding and correlate variables considered for final models were both time variant and time invariant and included relevant socio-demographic characteristics and aspects of FSWs’ living situation and work environments.

Time-variant covariates included in final adjusted models were marital/cohabitation status, average monthly income, alcohol consumption, recent drug use, average number of clients per week, and perceived financial security. Marital/cohabitation status was defined as currently married or living with a sex partner vs. not. Average monthly income was defined as self-reported average monthly income, including sex work, over the last six months and dichotomized at 120,000 Tsh (median average monthly income at baseline; approximately $51USD) to compare higher vs. lower average monthly income. Alcohol consumption was measured using the AUDIT-3 short-form [44] and was based on aggregated scores across three questions assessing frequency of alcohol consumption per week, number of drinks consumed when drinking, and frequency of drinking six or more drinks on a single occasion. Summary scores were dichotomized into hazardous drinking (scores of three or more) and not hazardous drinking (scores of less than three). Drug use was measured based on self-reported use of any drugs within the past six months, including substances such as marijuana, khat, or heroin. Average number of clients per week was a binary variable dichotomized at the baseline median response, comparing an average of more than two clients per week to an average of two or fewer clients per week. Perceived financial security was self-reported and based on responses to the question “In the last six months, how would you describe your overall financial security?” This variable compared participants who reported “excellent, very good, good, or fair” to those who reported “poor.”

Time invariant covariates measured at baseline included participants’ age (categorized into four quartiles based on exploratory analyses: younger than 23, 23–25, 26–30, and over 30 years of age), HIV status (HIV-infected at baseline vs. not), community (intervention vs. control community), participants’ ethnic group (Hehe vs. other), and educational attainment (secondary school or higher vs. less than secondary school).

### Statistical analysis

Descriptive analyses examined recent experience of physical or sexual GBV, sex work-related mobility, and other covariates of interest at baseline and/or follow-up. Key baseline socio-demographic characteristics were compared between those individuals participating at baseline and follow-up (n = 387) and those not participating in the follow-up survey (n = 109) to examine potential effects of loss to follow-up using Pearson’s χ^2^ tests. To examine bivariate associations between sex work-related mobility as well as other covariates and the outcome of interest, Pearson’s χ^2^ tests were conducted to examine associations at baseline and at follow-up. As recent experience of physical or sexual GBV was commonly reported among study participants, unadjusted Poisson regression models were fit using generalized estimating equations (GEE) with robust variance estimations and an exchangeable correlation structure to examine the longitudinal relationship with sex work-related mobility as well as with other variables of interest. The use of robust Poisson regression was proposed as an alternative to logistic regression for common outcomes [45]. Longitudinal models accounted for clustering within FSWs across time points. All analyses were conducted in Stata15 [46].

It was hypothesized that age was a potential effect modifier of the relationship between sex work-related mobility and recent experience of physical or sexual GBV. An interaction term between the explanatory variable of interest and this socio-demographic characteristic was included in bivariate robust Poisson regression models to examine this hypothesis. There was no significant evidence of effect modification at the bivariate level (p>0.05), and the interaction term was dropped in final multivariate models presented here.

An adjusted robust Poisson regression model first included relevant factors related to FSWs’ socio-demographic characteristics, living situation, and work environments. A manual backward elimination method from this initial full robust Poisson model was conducted. Wald tests were used to assess overall contribution of variables to the final model. Variables were removed if they did not significantly contribute to the model assessed based on p>0.2. Following this step, retained covariates were included in the final model examining the longitudinal relationship between sex work-related mobility and recent experience of physical or sexual GBV. As part of a sensitivity analysis, the final model suggested following this manual backward selection approach was compared to models suggested using forward and backward stepwise selection with a cut-off of p = 0.2. Model fit was assessed using Cui’s quasilikelihood under the independence model criterion (QIC), an adaptation of Akaike’s information criterion (AIC) for use with GEE analyses, to identify the best group of covariates for the final model [47]. The final model presented here had the lowest QIC among adjusted models fit. Variance inflation factors (VIFs) were calculated to assess collinearity of explanatory variables. All VIFs were less than two, suggesting minimal collinearity of covariates.

The final model adjusted for study visit (follow-up vs. baseline), community, HIV status at baseline, age, educational attainment at baseline, ethnic group, marital/cohabitation status, average number of clients per week, perceived financial security, alcohol consumption, and recent drug use. All coefficients were exponentiated and reflect either unadjusted or adjusted incidence rate ratios (IRR).

## Results

Table 1 shows sex work-related mobility, socio-demographic characteristics, living situation, and work environment-related variables by recent experience of physical or sexual GBV at baseline and follow-up. Among 387 FSWs participating in both baseline and follow-up surveys, 40% of FSWs at baseline and 29% at follow-up reported experiencing physical or sexual violence from a client or partner in the six months prior to the survey. Sex work-related mobility within the past six months was reported by 26% and 11% of FSWs at baseline and follow-up respectively. Among FSWs mobile for sex work at follow-up (n = 42), 71% reported traveling once a month or less, and 29% reported traveling several times a month or more frequently. Forty-four percent of FSWs (n = 171) were HIV-positive at baseline.

**Table 1:** Recent sex work-related mobility, socio-demographic characteristics, living situation, and work environment by recent experience of gender-based violence (GBV; physical or sexual violence) at baseline and follow-up among female sex workers (FSWs) who participated at both baseline and follow-up (n = 387).

	Baseline GBV							Follow-up GBV						
	No		Yes		Total			No		Yes		Total		
	(n)	(%)	(n)	(%)	(n)	(%)	p-value^1^	(n)	(%)	(n)	(%)	(n)	(%)	p-value^1^
Sex work-related travel outside Iringa, last six months^2^							p = 0.008**							p = 0.043*
None	182	78.8	104	66.7	286	73.9		247	91.1	95	84.1	342	89.1	
Any	49	21.2	52	33.3	101	26.1		24	8.9	18	15.9	42	10.9	
**Community**							p = 0.303							p = 0.278
Control community	121	52.4	90	57.7	211	54.5		144	52.7	67	58.8	211	54.5	
Intervention community	110	47.6	66	42.3	176	45.5		129	47.3	47	41.2	176	45.5	
**HIV status (baseline)**							p = 0.522							p = 0.758
Negative	132	57.1	84	53.8	216	55.8		151	55.3	65	57.0	216	55.8	
Positive	99	42.9	72	46.2	171	44.2		122	44.7	49	43.0	171	44.2	
**Age (baseline)**							p = 0.486							p = 0.015*
≤22 years	50	21.6	38	24.4	88	22.7		59	21.6	29	25.4	88	22.7	
23–25 years	54	23.4	30	19.2	84	21.7		67	24.5	17	14.9	84	21.7	
26–30 years	59	25.5	48	30.8	107	27.6		65	23.8	42	36.8	107	27.6	
>30 years	68	29.4	40	25.6	108	27.9		82	30.0	26	22.8	108	27.9	
**Educational attainment (baseline)**							p = 0.054^							p = 0.248
Less than secondary school	172	74.5	102	65.4	274	70.8		198	72.5	76	66.7	274	70.8	
Any secondary school or more	59	25.5	54	34.6	113	29.2		75	27.5	38	33.3	113	29.2	
**Ethnic group (baseline)**							p = 0.916							p = 0.025*
Other	136	58.9	91	58.3	227	58.7		170	62.3	57	50.0	227	58.7	
Hehe	95	41.1	65	41.7	160	41.3		103	37.7	57	50.0	160	41.3	
**Marital/cohabitation status**							p = 0.498							p = 0.015*
Not currently married/living with sex partner	163	70.6	115	73.7	278	71.8		184	67.4	62	54.4	246	63.6	
Currently married/living with sex partner	68	29.4	41	26.3	109	28.2		89	32.6	52	45.6	141	36.4	
**Average monthly income, past six months**							p = 0.068^							p = 0.036*
≤ 120000Tsh	127	55.0	71	45.5	198	51.2		168	61.5	57	50.0	225	58.1	
>120000Tsh	104	45.0	85	54.5	189	48.8		105	38.5	57	50.0	162	41.9	
Perceived financial security^3^							p = 0.287							p = 0.169
Poor	65	28.3	52	33.3	117	30.3		99	36.4	50	43.9	149	38.6	
Fair, good, very good, or excellent	165	71.7	104	66.7	269	69.7		173	63.6	64	56.1	237	61.4	
Average number of clients per week^4^							p = 0.002**							p = 0.008**
2 or fewer	159	68.8	83	53.2	242	62.5		209	76.8	72	63.7	281	73.0	
More than 2	72	31.2	73	46.8	145	37.5		63	23.2	41	36.3	104	27.0	
**Alcohol consumption**							p = 0.045*							p<0.0001***
Not hazardous	60	26.0	27	17.3	87	22.5		131	48.0	25	21.9	156	40.3	
Hazardous drinking	171	74.0	129	82.7	300	77.5		142	52.0	89	78.1	231	59.7	
**Any drug use in the past six months**							p = 0.004**							p = 0.614
None	229	99.1	147	94.2	376	97.2		268	98.2	111	97.4	379	97.9	
Any	2	0.9	9	5.8	11	2.8		5	1.8	3	2.6	8	2.1	
**Total**	231	100.0	156	100.0	387	100.0	--	273	100.0	114	100.0	387	100.0	--

^1^ Reported p-values based on Pearson’s χ^2^ tests.

^2^ Missing responses at follow-up (n = 3)

^3^ Missing responses at baseline and follow-up (n = 1)

^4^ Missing responses at follow-up (n = 2).

*** p<0.001

** p<0.01

* p<0.05

^ p<0.1.

There were no significant differences in the outcome or primary explanatory variables of interest at baseline between the final sample (n = 387) and those who did not participate in the follow-up survey (n = 109). Those lost to follow-up tended to be younger, not living with HIV, and not currently married or living with a sex partner.

At baseline and follow-up, mobility for sex work was more common among FSWs reporting recent physical or sexual violence from a client or partner than among those with no recent experience of violence (baseline: 33% vs. 21%; follow-up: 16% vs. 9%; Table 1). In unadjusted longitudinal models, FSWs who had recently traveled outside of Iringa in the last six months primarily for sex work had a 1.49 times increased risk of reporting recent experience of physical or sexual GBV as compared to those with no recent sex work-related mobility (95% CI: 1.22–1.83; p<0.001; Table 2). Among socio-demographic, living situation, and work environment-related variables, higher educational attainment (unadjusted IRR: 1.25; 95% CI: 1.01–1.56; p<0.05) and higher average monthly income within the past six months (unadjusted IRR: 1.32; 95% CI: 1.09–1.61; p<0.01) were positively associated with recent experience of physical or sexual GBV. Reporting more than two clients on average per week (unadjusted IRR: 1.47; 95% CI: 1.21–1.78; p<0.001), hazardous alcohol consumption (unadjusted IRR: 1.89; 95% CI: 1.48–2.41; p<0.001), and any drug use in the past six months (unadjusted IRR: 1.78; 95% CI: 1.23–2.57; p<0.01) were significantly associated with an increased risk of reporting recent experience of physical or sexual GBV as compared to those with two or fewer clients per week, non-hazardous alcohol consumption, or no recent drug use respectively (Table 2).

**Table 2:** Bivariate and multivariate analyses of longitudinal associations between mobility and experience of any gender-based violence (GBV; physical or sexual) in the last six months among female sex workers (FSWs) who participated at both baseline and follow-up (n = 387).

	Any physical or sexual GBV in last six months	
VARIABLES	*Unadjusted IRR (95% CI)*	*Adjusted IRR (95% CI)*
**Travelled outside Iringa in last six months for sex work** (Any vs. none) ^#^^,^ ^$^	1.49***	1.25*
	(1.22–1.83)	(1.03–1.53)
**Visit** (Follow-up vs. baseline)	0.73***	0.81*
	(0.62–0.87)	(0.67–0.97)
**Community** (Intervention community vs. control community)	0.86	0.85
	(0.69–1.08)	(0.68–1.06)
**HIV status (baseline)** (HIV-infected at baseline vs. not)	1.03	1.12
	(0.83–1.27)	(0.91–1.39)
**Age (baseline)** (Ref: ≤22 years)		
23–25 years	0.73^	0.71*
	(0.53–1.02)	(0.52–0.98)
26–30 years	1.10	1.00
	(0.84–1.45)	(0.77–1.30)
>30 years	0.80	0.73^
	(0.59–1.09)	(0.52–1.01)
**Educational attainment (baseline)** (Any secondary school or higher vs. less)	1.25*	1.24^
	(1.01–1.56)	(1.00–1.55)
**Ethnic group (baseline)** (Hehe vs. not)	1.17	1.18
	(0.94–1.45)	(0.95–1.47)
**Marital/cohabitation status** (Currently married/living with sex partner vs. not) ^#^	1.10	1.33**
	(0.90–1.35)	(1.09–1.63)
**Average monthly income, past six months** (>120000 Tsh vs. ≤120000 Tsh) ^#^^,^ ^$^	1.32**	1.21^
	(1.09–1.61)	(0.99–1.48)
**Perceived financial security in last six months** (Poor vs. fair, good, very good, or excellent) ^#^^,^ ^$^	0.86	0.79*
	(0.71–1.04)	(0.65–0.96)
**Average number of clients per week** (>2 vs. ≤2) ^#^	1.47***	1.28*
	(1.21–1.78)	(1.05–1.56)
**Alcohol consumption** (Hazardous vs. not hazardous) ^#^	1.89***	1.64***
	(1.48–2.41)	(1.28–2.12)
**Any drug use in the past six months** (any vs. none) ^#^^,^ ^$^	1.78**	1.39*
	(1.23–2.57)	(1.03–1.87)
Observations	--	767
Number of participants	387	387

IRR: Incidence rate ratio.

CI: Confidence interval.

Robust 95% CI in parentheses.

*** p<0.001

** p<0.01

* p<0.05

^ p<0.1.

^#^ Time-variant explanatory variables.

^$^ variable refers specifically to the last six months.

In the final adjusted longitudinal model, sex work-related mobility outside of Iringa in the past six months remained significantly and positively associated with any physical or sexual GBV. Recent sex work-related mobility was associated with a 25% increased risk of recent experience of physical or sexual GBV when compared with no recent reported mobility for sex work (adjusted IRR: 1.25; 95% CI: 1.03–1.53; p<0.05). Age, educational attainment, and average monthly income were marginally significantly associated with recent experience of physical or sexual GBV in the final adjusted model (Table 2). Participants who were currently married or living with a sex partner had, in the adjusted model, a 33% increased risk of recent experience of physical or sexual GBV when compared to those not married or living with a partner (adjusted IRR: 1.33; 95% CI: 1.09–1.63; p<0.01). Those reporting greater financial security had significantly lower risk of recently experiencing physical or sexual GBV as compared to those reporting poor financial security in the last six months (adjusted IRR: 0.79; 95% CI: 0.65–0.96; p<0.05). Average number of clients per week (adjusted IRR: 1.28; 95% CI: 1.05–1.56; p<0.05), alcohol consumption (adjusted IRR: 1.64; 95% CI: 1.28–2.12; p<0.001), and drug use (adjusted IRR: 1.39; 95% CI: 1.03–1.87; p<0.05) all remained significantly positively associated with recent experience of physical or sexual GBV in adjusted models (Table 2).

## Discussion

In this sample of FSWs from Iringa, Tanzania, FSWs with recent mobility for sex work had a higher risk of recent physical or sexual GBV when compared to those with no recent mobility for sex work after adjusting for socio-demographic characteristics and aspects of their living situations and work environments. Other aspects of FSWs’ living situations and work environments, such as FSWs’ self-reported average number of clients per week, hazardous alcohol consumption, and recent drug use, also had positive associations with any recent experience of physical or sexual GBV. This study, consistent with previous analyses conducted in Vancouver, Canada [16], strengthens our understanding of the longitudinal association between FSWs’ mobility and violence in Sub-Saharan Africa. Previous research in India has demonstrated a high prevalence of recent experiences of violence among mobile FSWs [6], but previous studies outside of North America have failed to explore these associations longitudinally [25–28]. This article provides urgently needed evidence outside of North America using longitudinal data of the relationship between sex work-related mobility and risk of GBV for FSWs.

While addressing the factors that put FSWs at greater risk of GBV is increasingly a priority for public health interventions, these findings suggest that there is a need to acknowledge the ways in which mobile FSWs, particularly those mobile for the purposes of sex work, may experience unique risk environments that increase their risk for and experiences of GBV. Participatory involvement of mobile FSWs in the conceptualization and design of interventions would not only strengthen engagement, but also address the unique needs that mobile FSWs have while traveling. This could include, for example, mobile GBV services or the improved integration of GBV services into other mobile health services or outlets providing services to FSWs. These services could address both prevention and response and include social support, case management, and referrals. Lessons could be learned from those working in other settings at the intersection of substance use, sex work, and mobility [48], for how best to implement such a program. Interventions to improve FSWs’ ability to access care and treatment at multiple locations when traveling, including HIV-related care and treatment for FSWs living with HIV, could be coupled with interventions designed to strengthen health providers’ capacity to recognize and respond to GBV. Finally, mobile or digital intervention tools [49] could improve FSWs’ long-term engagement in community-based interventions, connect FSWs to support services when traveling, facilitate connections between FSWs to foster social cohesion, or serve as a resource for tips and information for how to prevent GBV during trips. With mobile phones increasingly accessible to and used by FSWs in Iringa, text- or interactive voice response (IVR)-based messaging could be a useful strategy. However, mobility must be addressed in tandem with other aspects of FSWs’ work environments, such as substance use [13,42], that also were found to be significantly associated with recent experience of sexual or physical GBV in this study.

Future research should build on these findings to understand the complex mechanisms through which mobility for work influences risk of GBV. A qualitative investigation, for example, of the drivers of mobility for work in Tanzania would illuminate how mobility for work influences FSWs’ risk environments. Quantitative studies could also build on the findings presented here to explore those socio-structural factors–including factors such as alcohol consumption, drug use, client interactions, or other aspects of FSWs’ risk environments–that may mediate the relationship between work-related mobility and recent experiences of GBV. In addition, while there was no significant evidence of effect modification by age, mobility and recent experiences of GBV were more common among select age groups of FSWs, and further quantitative research is needed to understand these dynamics including whether or through what pathways age and mobility influence FSWs’ risk of GBV. Studies have previously looked at the intersection of mobility and GBV and its effect on other health outcomes like depression [50] or HIV infection [25]. Together, such explorations would provide insights into the pathways and mechanisms through which mobility and GBV are linked, which could then enable public health interventions to be more sensitive to the unique needs and lived realities of mobile FSWs. However, a focus on these downstream pathways should be done in tandem with efforts to address the social determinants of health [51] that influence not only FSWs’ mobility for work, but also perpetuate the gender inequalities and stigma and discrimination that coincide [13,42] to put FSWs at increased risk of violence from clients and partners.

This study reinforces the connection between mobility and gender inequality, made manifest in instances of GBV [52], in our conceptual understanding of FSWs’ lived realities and risk environments in Tanzania. Theoretical and conceptual linkages between gender and population mobility [53,54] have typically emphasized how gender and migration are mutually constitutive. As Haram (2004) wrote of women in Northern Tanzania, “geographic mobility involves, particularly for women, much more than a shift in physical location” [55]. Gender inequalities and norms influence whether and how individuals migrate, how they are perceived by others, and their experiences at place of origin, in transit, and at their destination. At the same time, such mobility has the potential to reinforce and challenge those gender inequalities. For FSWs, this interplay must also be understood within the context of the stigma and discrimination of sex work, with GBV not only the manifestation of and consequence of gender inequality, but also enacted stigma [1,42]. Given the intimate linkages between population mobility, GBV, and HIV [13,56], it is imperative that efforts to understand and intervene in HIV risk environments for FSWs acknowledge and complicate their frameworks to incorporate a more nuanced role for population mobility and GBV.

### Limitations

This study extends previous cross-sectional analyses [21] to examine longitudinal correlates of any recent experience of GBV. First, analyses presented here include data collected from two time points, with FSWs lost to follow-up excluded from analyses. As a result, it is possible that those FSWs who dropped out may have been different from those who remained in the study. While there were no significant differences in the outcome or explanatory variables of interest at baseline between those who participated in both baseline and follow-up and those who did not, those who were lost to follow-up tended to be younger, not living with HIV, and not married/living with a sex partner at baseline. As mobility is often correlated with age and is a common reason for participants being lost to follow-up and a common challenge for surveillance [12], it is likely that the estimates presented here underestimate recent mobility for sex work among FSWs in Iringa at follow-up. Future research should examine the decline in sex work-related mobility from baseline to follow-up (26% to 11%) to understand the factors, be it contextual (e.g. seasonal variations in mobility patterns) or related to participation, engagement, and retention in the parent study over time, that may influence such mobility.

Second, this study is limited by its measures of GBV and FSWs’ mobility. Measures of GBV at baseline and follow-up limited analyses to any recent experience of GBV from a client or partner. Future research should ensure consistent measurement of GBV across time points, and may need to include GBV from other perpetrators, such as police or community members. In addition, there remains a need for future research to understand the nuanced relationships between mobility and GBV. Recent work in Zimbabwe has begun to identify typologies of mobility based on destination, duration, and other aspects of FSWs’ mobility [57]. Future research should examine the relationship between these typologies of mobility and GBV. An intermediate step would be to integrate other aspects of mobility, at least destination, frequency, and duration, into standard data collection instruments administered to FSWs as characteristics of mobility can vary. A qualitative investigation of mobility trajectories could elaborate on or identify emergent mobility typologies for FSWs. Previous research in north-western Tanzania, for example, used qualitative, ethnographic methods to identify typologies of women and men living and working in a mining community that described their unique risks for HIV and other infections [34]. These typologies illustrated how FSWs’ mobility was often circular, following the pay periods of those working in the mines. The expansion of such an approach to exploring mobility among FSWs in greater depth could be used to understand the mechanisms by which mobility and GBV intersect in FSWs’ lives.

Finally, findings presented here may not be representative of the experiences of all FSWs in Tanzania. Analyses were conducted using baseline and follow-up data from Project Shikamana, a community-randomized trial conducted in two communities in Iringa. While Project Shikamana included activities focused on violence prevention, there were no significant reductions in violence found in adjusted models, suggesting the need for more comprehensive approaches that address the socio-structural factors that influence GBV in this context. The socio-structural environment and context in communities where this study was conducted may differ from other communities in Iringa and elsewhere in Tanzania, given their proximity to the Tan-Zam highway [31] that may influence not only FSWs’ mobility, but also their work environments.

## Conclusions

Drawing on baseline and follow-up data from a community randomized controlled trial in Iringa, Tanzania, this study examined the longitudinal relationship between recent mobility for the purposes of sex work and experiences of GBV from a client or partner. Evidence of a significant increased risk of violence among mobile FSWs as compared to their non-mobile counterparts suggests that efforts to reduce GBV among FSWs must acknowledge the unique needs of those FSWs who are mobile for sex work. Public health programs must identify ways–using digital technologies, for example–to engage mobile FSWs. Further understanding of the typologies of FSW mobility that integrate a qualitative understanding of FSWs’ trajectories with quantitative understandings of not only reason for mobility but also factors related to destination, duration, and frequency will help elucidate the pathways through which mobility influences FSWs’ risk environments and, ultimately, their experiences of violence. An integration of mobility into how we understand FSWs’ risk environments, building on the strong body of literature exploring the intersections between migration and gender, is a critical first step for those working with such mobile populations.
